# Patients’ level of satisfaction on quality of health care at Mwananyamala hospital in Dar es Salaam, Tanzania

**DOI:** 10.1186/1472-6963-14-400

**Published:** 2014-09-18

**Authors:** Kudra Khamis, Bernard Njau

**Affiliations:** Kilimanjaro Christian Medical University College, Box 2240, Moshi, Tanzania; Kilimanjaro Christian Medical Centre, Box 3010, Moshi, Tanzania

**Keywords:** Patient satisfaction, Quality of care, Donabedian model, Tanzania

## Abstract

**Background:**

Enhancing quality of health care delivered in public health facilities in developing countries is a key prerequisite to increase utilization and sustainability of health care services in the population. The aim of the study was to determine patients’ level of satisfaction on the quality of health care delivered at the out-patient department (OPD) in Mwananyamala hospital in Dar es Salaam, Tanzania.

**Methods:**

A cross-sectional study design was conducted from April to May, 2012. A systematic sampling method was employed to select 422 study subjects. A pre-tested SERVQUAL questionnaire was used to collect data and one-sample t-test was employed to identify patients’ level of satisfaction and principal component analysis to identify key items that measure quality of care.

**Results:**

Patients’ level of satisfaction mean gap score was (-2.88 ± 3.1) indicating overall dissatisfaction with the quality of care. The level of dissatisfaction in the five service dimensions were as follows: assurance (-0.47), reliability (-0.49), tangible (-0.52), empathy (-0.55), and responsiveness (-0.72).

**Conclusion:**

Patients attending OPD at Mwananyamala hospital demonstrates an overall dissatisfaction on quality of care. Hospital management should focus on: improvement on communication skills among OPD staff in showing compassion, politeness and active listening, ensure availability of essential drugs, and improvement on clinicians’ prescription skills.

## Background

Quality of health care is defined as a degree of performance in relation to a defined standard of interventions known to be safe and have the capacity to improve health within available resources [1].

The patient satisfaction perspective of hospital care had gained more attention in recent years and studies have shown that patients are most satisfied with interpersonal interactions, such as staff-patient relationships [2]. A study done in South Africa concluded that patient satisfaction is a fundamental indicator of equitable quality of care [3]. Another study on patients’ satisfaction at a referral hospital in Tanzania observed a high level of satisfaction among respondents, mainly because of the hierarchical health care delivery system, whereby the referral hospital is at the apex with super-specialty services. However, a small proportion of patients were dissatisfied with long waiting time, high cost of treatment, and investigation charges [4]. It is well documented that if patients’ level of satisfaction on quality of care does not meet their standard, they may decide to seek for treatment somewhere else [5–7].

In fact, satisfied patients are likely to exhibit favorable behavioral intentions, which are beneficial to the healthcare provider’s long-term success. However, one of the major barriers to better health care for much of the population in developing countries, including Tanzania, is lack of access to even basic health services [8]. In Tanzania, despite efforts by the Government, through the Ministry of Health and Social Welfare (MoHSW), to improve the quality of care through different approaches such as Health Quality Improvement Framework, still health service provision is constrained by a number of factors in terms of poor infrastructure, unavailability of drugs and/or medical equipments and limited human resource for health [9, 10]. For example, in 2006 the national average population/doctor ratio was 138,000 persons per doctor, while the national average population/nurse ratio was 5,000 persons per nurse [11].

In Tanzania, 80% of all patients attending health facilities are attended at out-patient-department (OPD), hence making OPD a key area to assess quality of care [9–11]. To understand patients’ level of satisfaction it was imperative to conduct this study to determine patients’ level of satisfaction on the quality of health care delivered at the OPD in Mwananyamala hospital in Dar es Salaam, Tanzania.

This study used the Donabedian model [12] to determine patients’ level of satisfaction on quality of care at the study setting. According to the Donabedian model, three key domains, namely structure, process and outcome are interrelated in the context of quality of care. Donabedian posit that a good structure increases the likelihood of good process, which increases the likelihood of good outcome, such as patients’ satisfaction [12]. The Donabedian model was adopted in this study because it has received substantial empirical support for its ability to generate information from which inferences can be drawn on quality of care [12].

However, there is dearth of empirical evidence on the use of Donabedian model to assess patients’ satisfaction on quality of care provided at different health care facilities in Tanzania. This study therefore aims to determine patients’ level of satisfaction in an urban health care facility. Findings from this study will add knowledge to the literature by assessing how Donabedian model might explain patients’ level of satisfaction on quality of care and provide evidence for improvement of quality of care in the study setting.

## Methods

### Design and study area

A cross-sectional study design was conducted at Mwananyamala hospital from April to May 2012. Mwanayamala public hospital is located in Kinondoni municipality in Dar es Salaam, Tanzania. During the study period, Mwananyamala hospital use to attend 1500 to 1700 patients per day through six units at the OPD [13].

### Study population and sampling

A single population proportion sample size determination formula was used with the following assumption: the patients’ level of satisfaction in Dar es Salaam of 50% [14], margin error of 5%, and non response rate of 10% and the desired level of confidence interval at 95% [14]. A minimum sample size of 422 was calculated. A systematic sampling based on the projected daily attendance at OPD per day and a list of attending patients obtained from the medical records was used to select participants to participate in the exit interviews. To get the sampling interval a formula N/n was used whereby N = the total number of patients attending OPD per day, and n = the estimated sample size [14].

Anonymous, structured SERVIQUAL questionnaire was adapted and then adopted to address the study objectives [15]. The SERVIQUAL questionnaire is divided into five service dimensions (tangibles, reliability, responsiveness, assurance and empathy) to determine patients’ level of satisfaction on quality of care. According to SERVIQUAL questionnaire, the questions to assess patients’ level of satisfaction are in two categories: 1) expectation and 2) perception questions.

The questionnaire was developed in English with back-and-forth translated in Kiswahili-the local language in Tanzania. The SERVIQAUL questionnaire in Kiswahili was then piloted with a convenient sample of n = 30 (15 males vs. 15 females patients) for validity and reliability. Minor adjustments were made based on the pilot testing. The respondents were informed of the purpose of the study and assured of confidentiality and their right to withdraw from the study. Data was collected for 14 days with an average of 30 exit interviews per day by three trained research assistants.

### Study variables

The dependent variable in this study was Patients’ level of satisfaction and was assessed by asking the level to which they were satisfied with the structure and process domains using a four point-Likert Scale questions (rating points on the scale).

The explanatory variables in this study includes: socio demographic characteristics (sex, age, religion, marital status, occupation); Tangibles: Five questions were used to assess the tangibles variable. An example of expectation question was: “I expect drugs for all diseases to be available at the OPD”. Expected response was: 1 = strongly disagree; 4 = strongly agree. An example of perception question was: “I am satisfied that all drugs for all diseases are available at the OPD”. Expected response was: 1 = strongly disagree; 4 = strongly agree. The reliability scale was Cronbach’s alpha coefficients = .85.

Reliability: Four questions were used to assess the reliability variable. An example of expectation was: “I expect staff at the OPD to keep my appointments”. Expected response was: 1 = strongly disagree; 4 = strongly agree. An example of perception question was: “I am satisfied that staff at OPD kept my appointment”. Expected response was: 1 = strongly disagree; 4 = strongly agree. This scale was reliable at alpha = .76.

Responsiveness: Seven questions were used to assess the responsiveness variable. An example of expectation question was: “I expect staff at OPD to always retrieve my records promptly whenever required”. Expected response was: 1 = strongly disagree; 4 = strongly agree. An example of perception question was: “I am satisfied that staff at OPD retrieved my records promptly whenever required”. Expected response was: 1 = strongly disagree; 4 = strongly agree. The reliability scale was alpha = .80.

Assurance: Five questions were used to assess the assurance variable. An example of expectation question was: “I expect laboratory results at the OPD will be timely delivered”. Expected response was: 1 = strongly disagree; 4 = strongly agree. An example of perception question was: “I am satisfied that my laboratory results were timely delivered”. Expected response was: 1 = strongly disagree; 4 = strongly agree. This scale was reliable at alpha = .77.

Empathy: Five questions were used to assess the empathy variable. An example of expectation question was: “I expect staff at the OPD to pay attention to my medical concerns”. Expected response was: 1 = strongly disagree; 4 = strongly agree. An example of perception question was: “I am satisfied that staff at OPD paid attention to my medical concerns”. Expected response was: 1 = strongly disagree; 4 = strongly agree. The reliability scale was alpha = .83.

### Data management and analysis

Data was entered and cleaned in EPI INFO software and analyzed using STATA version 13.1 and Statistical Package for Social Sciences (SPSS) version 14.1. Descriptive as well as analytical analysis was employed to determine patients’ level of satisfaction. To calculate the mean gap score for patients’ level of satisfaction the following procedures was used. A total score (in%) was calculated for each dimension (e.g. Tangibles) for both expectation and perception questions. The total gap score was derived by subtracting perception score (%) from expectation score (%). A one-sample t-test was conducted to assess whether the two scores are statistically different from each other. The gap implies the level of patients’ satisfaction on quality of care. Quality of care is deemed indifferent or sufficient when patients’ level of satisfaction is equal or greater than the expected level of service or vice versa [15].

In addition, principal component analysis (PCA) was done using SPSS to identify the subgroups of SERVIQUAL items forming subscales. Prior to performing PCA, the suitability of data was assessed. Correlation coefficient was set at a cut of point of .3 or above. The Kaiser-Meyer-Oklin [16, 17] value-which was used to assess sampling adequacy was set at a cut-off point of .6, while the Bartlett’s test of sphericity [18], was used to support the factorability of the correlation matrix. Furthermore, a Catell’s scree test [19], and eigenvalue of over 1.0, which represents the amount of the total variance explained by a factor, were used to inspect the plotting of each eigenvalue of the factors to find a point at which the shape of the curve changes direction and becomes horizontal. All factors above the break in the plot and with eigenvalues of over 1.0 were retained for further analysis. Lastly, further analysis was done using Varimax method, to try to minimize the number of variables with high loadings on each factor.

### Ethical consideration

Ethical clearance was obtained from Kilimanjaro Christian Medical University College Ethics Committee. All respondents consented to take part in the study and ethical procedures were adhered to.

## Results

A total of 422 study participants were included with a response rate of 100%. Mean age of participants was 36.8 ± 11.9. Almost half, (n = 191, 45.3%) were aged 34 years or less, most, 52.8% were female, 55% were Christians and 84.6% were either employed or self-employed. Table 1 below summarize socio-demographic characteristics of all study participants.

**Table 1:** **Characteristics of participants**

Demographic characteristics	Sex of respondent (%)		Total
	Male	Female	
All respondents	199 (47.2)	223 (52.8)	422
**Religion**			
Christian	110 (55.3)	122 (54.7)	232 (55.0)
Muslim	89 (44.7)	101 (45.3)	190 (45.0)
**Occupation**			
Employed	85 (42.7)	93 (41.7)	178 (42.2)
Self employed	90 (45.2)	89 (39.9)	179 (42.4)
Unemployed	24 (12.1)	41 (18.4)	65 (15.4)
**Marital status**			
Married	92 (46.2)	106 (47.6)	198 (46.9)
Single	73 (36.7)	75 (33.6)	148 (35.1)
Widowed	13 (6.5)	21 (9.4)	34 (8.1)
Divorced	21 (10.6)	21 (9.4)	42 (10.0)
**Age in years**			
≤ 34 yrs	88 (44.2)	103 (46.2)	191 (45.3)
35-54 yrs	98 (49.2)	97 (43.5)	189 (44.8)
≥55 yrs	19 (6.6)	23 (10.3)	42 (10.0)

### Overall patients’ level of satisfaction

**Figure 1:**
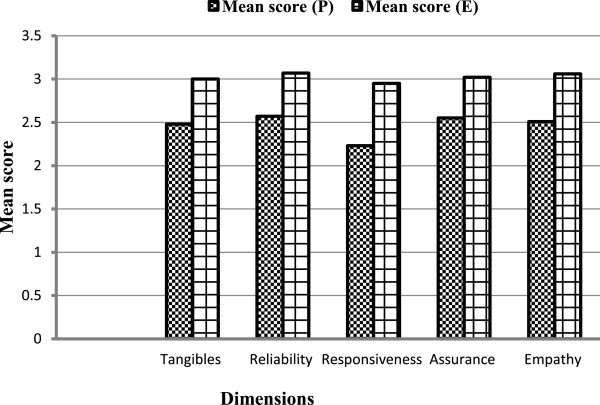
**Mean score for 5 service dimensions to assess quality of care.**

Overall, the mean gap score (standard deviation) to assess overall patients’ level of satisfaction attending OPD at Mwananyamala hospital was relatively small – 2.88 (±3.1). The mean expectation score was 15.1 while the mean perception score was 12.2. Therefore, the gap mean score (Perception - Expectation) was -2.88 of all five service dimensions assessed.Figure 1 below shows a summary of mean score of five service dimensions including tangibles, responsiveness, reliability, assurance and empathy.

### Ranking of service dimensions to assess quality of care

Of the five service dimensions, assurance was ranked first. Expectation score for assurance was 3.02 and standard error (SE) 0.143, while the mean perception score was 2.55 and SE 0.151, therefore the gap mean score for assurance was -0.47. Of all five items used to assess assurance, respondents were least likely being satisfied with confidentiality of patient’s records at the OPD (-0.20; SE = 0.041; p < 0.001), less likely to recommend OPD service to other patients (-0.43; SE = 0.039; p < 0.001), least satisfied by the skills and knowledge of staff to answer questions (-0.47; SE = 0.039; p < 0.001), timely availability of laboratory results (-0.54; SE = 0.045; p < 0.001), and availability of adequate staff at OPD (-0.69; SE = 0.045; p < 0.001).

Reliability was ranked second. Expectation score for reliability was 3.07 (SE = 0.10) while the mean perception score was 2.57 (SE = 0.11), therefore the gap mean score for assurance was -0.49. Of all four items used to assess reliability, respondents were least satisfied with proper prescription of medications (-0.35; SE = 0.047; p < 0.001), communication skills of the staff (-0.5; SE = 0.040; p < 0.001), on how staff kept their appointments (-0.55; SE = 0.043; p < 0.001), and physical examination of clients (-0.61; SE = 0.043; p < 0.001). Tangible was ranked third. The mean expectation score for tangibles was 3.00 (SE = 0.143), while the mean perception score was 2.48 (SE = 0.151), therefore the gap mean score for tangibles was -0.52. Of all five items used to assess tangibles dimension, respondents were least satisfied with OPD clinicians ability to prescribe good drugs (-0.41; SE = 0.044; p < 0.001), availability of drugs for all diseases (-0.43; SE = 0.045; p < 0.001), general cleanliness at OPD (-0.50; SE =0.045; p < 0.001), accessibility of drugs in the OPD (-0.57; SE = 0.049; p < 0.001), and sufficient chairs and toilets at OPD (-0.67; SE = 0.047; p < 0.001).

Empathy was ranked fourth. Mean expectation score for empathy was 3.06 (SE = 0.120), while the mean perception score was 2.51 (SE =0.140), therefore the gap mean score for assurance was -0.55. Of all five items used to assess empathy, respondents were least satisfied with compassion (-0.50; SE=; 0.038; p < 0.001), active listening to patients (-0.51; SE=; 0.036; p < 0.001), politeness, comforting and encouraging when patients face medical problems (-0.55;SE=; 0.037; p < 0.001), OPD staff paid attention to individual medical concerns of patients (-0.60; SE = 0.043; p < 0.001), and OPD staff built good cooperation with patients (-0.62; SE = 0.041; p < 0.001).

Responsiveness was ranked fifth. Mean expectation score for responsiveness was 2.95 (SE = 0.195) while the mean perception score was 2.23 (SE = 0.203), therefore the gap mean score for assurance was -0.72. Of all seven items used to assess responsiveness, respondents were least satisfied with OPD staff respect of patients (-0.42; SE = 0.041; p < 0.001), ability of OPD staff to assist when medical help is needed (-0.46; SE = 0.040; p < 0.001), OPD staff offer prompt services (-0.51; SE = 0.043; p < 0.001), time taken by OPD staff when attending patients’ problems (-0.51; SE = 0.042; p < 0.001), prompt retrieval of patients’ records (-0.53; SE = 0.043; p < 0.001), waiting time of patients before getting services (-0.56; SE = 0.041; p < 0.001) and identify very ill patients and offer help (-0.64; SE = 0.040; p < 0.001). Table 2 below summarizes the mean score on perception score and expectation score and ranking of the five service dimensions which assessed quality of care.

**Table 2:** **Mean score and ranking of 5 service dimensions to assess quality of care (n = 422)**

SN	SERVQUAL statements	Mean perception score (SE)	Mean score expectation (SE)	Mean gap score (SE)	P-value
**I**	**Tangibles (structure)**				
1	OPD has provided me with drugs of all diseases	2.64(.033)	3.07(.036)	-0.43(.045)	0.0000
2	Doctors of this OPD has prescribed good drugs	2.72(.031)	3.13(.037)	-0.41(.044)	0.0000
3	Drugs are obtained easily in this OPD	2.27(.037)	2.84(.037)	-0.57(.049)	0.0000
4	OPD has good reception area that have sufficient seats and toilets	2.26(.035)	2.93(.034)	-0.67(.047)	0.0000
5	OPD appears clean every day	2.53(.036)	3.03(.030)	-0.50(.045)	0.0000
	**Average Tangibles SERVQUAL scores**	**2.48(.151)**	**3.00(.143)**	**-0.515**	**3**
**II**	**Reliability (process)**				
6	OPD staff keeps appointments given to me.	2.39(.033)	2.94(.034)	-0.55(.043)	0.0000
7	OPD staff has good communication and information skills.	2.75(.030)	3.25(.033)	-0.50(.040)	0.0000
8	OPD staff has fulfilled my expectations by giving me thorough physical examinations.	2.57(.034)	3.17(.031)	-0.60(.043)	0.0000
9	OPD staff has given me proper medications as prescribed (essential drugs)	2.59(.035)	2.91(.032)	-0.35(.047)	0.0000
	**Average Reliability SERVQUAL scores**	**2.57(.110)**	**3.07(.100)**	**-0.494**	**2**
**III**	**Responsiveness (process)**				
10	OPD staff retrieves my records promptly.	2.08(.034)	2.61(.037)	-0.53(.043)	0.0000
11	OPD staff identifies very ill patients and assist them whenever there is need.	2.62(.031)	3.26(.029)	-0.64(.039)	0.0000
12	OPD staff is respectful to me.	2.75(.032)	3.17(.028)	-0.42(.041)	0.0000
13	OPD staff offer prompt services.	2.40(.034)	2.91(.032)	-0.51 (.043)	0.0000
14	OPD staff is willing to help client whenever medical help was needed	2.36(.032)	2.82(.033)	-0.46(.040)	0.0000
15	I used a short period of time to wait (<30 min) before getting services	2.40 (.036)	2.96(.030)	-0.56(.041)	0.0000
16	OPD staff spend enough time (at least 10 min) while attending to my problems	2.45(.035)	2.96(.032	-0.51(.042)	0.0000
	**Average Responsiveness SERVQUAL scores**	**2.23(.203)**	**2.95(.195)**	**-0.722**	**5**
**IV**	**Assurance (structure)**				
17	Laboratory results of this OPD are timely availed	2.33(.036)	2.87(.034)	-0.54(.045)	0.0000
18	OPD staff adhere to the confidentiality of my information	2.98(.034)	3.18(.030)	-0.20(.041)	0.0000
19	OPD has adequate staffs to take care of its clients	2.23(.034)	2.92(.033)	-0.69(.045)	0.0000
20	OPD staff has enough knowledge to answer my questions	2.63(.032)	3.10(.028)	-0.47(.039)	0.0000
21	I can recommend this OPD services to other client	2.58(.033)	3.01(.030)	-0.43(.039)	0.0000
	**Average Assurance SERVQUAL scores**	**2.55(.143)**	**3.02(.132)**	**-0.466**	**1**
**V**	**Empathy (process)**				
22	OPD staff paid attention to my individual medical concerns	2.36(.036)	2.96(.032)	-0.60(.043)	0.0000
23	OPD staff has built good cooperation with me and are ready to offer me medical assistance	2.34(.032)	2.95(.031)	-0.61(.041)	0.0000
24	OPD staff is polite, comforting and encouraging to me when faced with medical problems	2.56(.031)	3.11(.025)	-0.55(.037)	0.0000
25	OPD staffs were compassionate to me	2.64(.034)	3.13(.025)	-0.49(.038)	0.0000
26	OPD staff listened to me adequately	2.63(.033)	3.14(.024)	-0.51(.036)	0.0000
	**Average Empathy SERVQUAL scores**	**2.51(.140)**	**3.06(.120)**	**-0.554**	**4**

The 26 items of the SERVIQUAL scale were subjected to Principal Component Analysis (PCA). Prior to performing PCA the suitability of data for factor analysis was assessed. Inspection of correlation matrix revealed the presence of many coefficients of .3 and above. The Kaiser-Meyer-Oklin value was .87, exceeding the cut-off point of .6 and the Barlett’s Test of Sphericity was statistical significant (X^2^ = 2648 (66); p < 0.001). Principal component analysis for expectation scale revealed the presence of seven components with eingenvalues exceeding 1, explaining 31.3%, 8.7%, 7.0%, 6.5%, 5.2%, 4.5% and 4.0% of the variance. These seven components explained a total of 67.2% of the variance. Principal component analysis for perception scale revealed the presence of six components with eingenvalues exceeding 1, explaining 41.6%, 6.3%, 6.0%, 5.0%, 4.1% and 4.0%. These six components explained a total of 67% of the variance.

Using Catell’s scree test, two components above the break point on the screeplot of factors for both expectation and perception scale were retained for further analysis. Further analysis using Varimox method revealed strong loading of six factors with both components. The two factor solution explained a total of 53 per cent of the variance, with Component 1 contributing 27.7% and Component 2 contributing 25.3% and had acceptance reliability, as indicated by Cronbach’s coefficients of .78 for expectation sub scale and .83 for perception sub scale. Table 3 above provides a summary of the Principal Component Analysis(PCA) of the SERVIQUAL scale.

**Table 3:** **Varimax Rotation of Two Factor Solution for SERVQUAL Items**

Items	Component 1	Compenent 2
	Expectation	Perception
	score	score
1. OPD staffs were compassionate to me	.83	.86
2. OPD staffs are polite, comforting and encouraging to me when faced with medical problems.	.78	.80
3. OPD staffs listened to me adequately	.74	.86
4. OPD staff has provided me with drugs of all diseases	.65	.73
5. Clinicians at this OPD have prescribed good drugs	.60	.67
6. OPD reception has sufficient seats and toilets	.57	.44
% of variance explained	27.7%	25.3%
Cronbach’s alpha coefficients	.78	.83

## Discussion

The study findings indicate that the overall patients’ level of satisfaction on the quality of care at the OPD in Mwananyamala hospital was relatively low. This is in line with findings of patients’ satisfaction studies elsewhere [20, 21]. However, this is contrary to a study done in Muhimbili National Hospital in Dar es Salaam, whereby a high proportion of patients were satisfied with quality of care [22]. In fact, it is not generally proven that patients’ satisfaction is related to quality as reported by Leonard (2008) in a study done in Arusha Tanzania. In this study Leonard showed that, in the Tanzanian context, satisfaction is not directly associated with quality, but changes in quality do lead to changes in satisfaction, as patients notice improvements [5]. Of all five dimensions used to assess patient’s level of satisfaction, respondents were least satisfied with the assurance dimension, followed by reliability, tangibles, empathy, and responsiveness. According to Donabedian model [12] there is a strong relationship between all three domains of structure, process and outcome, which exist in the context of quality of care. Based on the Donabedian model assurance dimension was used to assess the structure category. Of all items used to assess assurance dimension, respondents were least satisfied with OPD staff adherence to confidentiality of patient’s information. The observed dissatisfaction of patients’ on providers’ lack of adherence to confidentiality of patient’s information underline the importance for the hospital management to strengthen adherence skills among OPD staff on confidentiality of patient’s information. It is well documented that patients who perceive lack of confidentiality on their medical information tend to seek care somewhere else [5–7, 23, 24].

In this study reliability dimension was ranked second by respondents. Reliability dimension was used to assess the process domain according to the Donabedian model. In the reliability dimension, respondents were least satisfied with proper prescription of medications. The observed level of dissatisfaction of respondents on proper prescription of medications at the OPD may raise concern regarding clinician’s ability to make proper diagnosis and treatment of common diseases [23–25].

Tangibles dimension, which was used to assess the structure domain was ranked third by respondents. Respondents were dissatisfied with clinician’s ability to prescribe good drugs. This observation substantiates respondent’s dissatisfaction on proper prescription of medication mentioned in reliability dimension above. In this study, empathy was used to assess the process domain. Respondents were dissatisfied with lack of compassion by the OPD staff. Provider’s behavior towards patients, such as politeness is an important predictor for patient satisfaction [4, 5, 26]. This observation, which is reported to be common in most public health facilities, force patients who can afford to pay for services to choose to go to private health facilities instead of seeking care at public health facilities [26–29]. It is important for the hospital management at Mwananyamala to encourage the health personnel to embrace staff-patient relationship, whereby the patient is viewed as a customer in order to improve the quality of care in this setting.

Responsiveness was used to assess the process domain as well. Respondents were least satisfied with OPD staff respect towards patients. It is well documented that patient’s perception of health care provider’s behaviour, such as respect influence their view’s towards quality of care [4–6].

From the Principal Component Analysis (PCA), six items (3 empathy items versus 3 tangibles items) explained 53 percent of the patient’s satisfaction scores on quality of care. Empathy items which explained most of the dissatisfaction on quality of care were failure to show compassion, lack of politeness and inadequate listening by OPD staff. Tangibles items which explained most of the dissatisfaction on quality of care were lack of essential drugs, poor prescription of drugs by clinicians and insufficient seats and toilets at OPD. Apart from perceived poor prescription by clinicians at OPD, the perceived lack of essential drugs observed by respondents is crucial, because availability of essential drugs is an important factor influencing patients’ level of satisfaction observed in several studies in other settings [21–25].

According to the Donabedian model, all three domains, structure, process, and outcome are interrelated and in order to improve quality of care, all must function well to achieve the expected outcome, such as patients’ satisfaction [12]. However, it is important to note that a multitude of factors influence patients’ level of satisfaction and quality of care hence caution should be taken into consideration while making conclusions regarding quality of care [5, 8, 29–34].

### Study limitation and strength

This study has several limitations. First, the study, which was cross-sectional in nature, is unable to identify the causality of the outcome of interest. Second, the patient satisfaction relied on self response of participants and did not assess their views to changes in technical quality. It is well documented that self-reporting is liable to response bias. Third, this study only assessed patients who attended at the OPD and did not assess the patient-doctor interactions and may not reflect the overall quality of care at the hospital. A further study, which will include all departments in the hospital, and which will assess the effect of patient-doctor interactions to detect changes in technical quality of care is warranted to determine the overall quality of care in this setting.

The strength of this study is based on the fact that the study used the Donabedian model, which has been tested in many studies on patient’s satisfaction and revealed significant results. In addition this study used SERVIQUAL questionnaire, which is a standardized tool to measure service quality applied in different settings.

## Conclusion

In conclusion, respondents in this study perceived low quality of care provided at the OPD in Mwananyamala hospital. Key areas of concern includes: improvement on communication skills in showing compassion, politeness and active listening, availability of essential drugs, and improvement on clinicians’ prescription skills. If policy can improve the reality on the ground, patients will notice, and hence indirectly improve their satisfaction.
